# Assessing the dimensionality of the CES-D using multi-dimensional multi-level Rasch models

**DOI:** 10.1371/journal.pone.0197908

**Published:** 2018-05-25

**Authors:** Rainer W. Alexandrowicz, Rebecca Jahn, Johannes Wancata

**Affiliations:** 1 Institute for Psychology, Alpen-Adria-Universität Klagenfurt, Klagenfurt, Austria; 2 Department of Psychiatry and Psychotherapy, Medical University of Vienna, Vienna, Austria; University of Illinois at Chicago College of Medicine, UNITED STATES

## Abstract

**Objectives:**

The CES-D is a widely used depression screening instrument. While numerous studies have analysed its psychometric properties using exploratory and various kinds of confirmatory factor analyses, only few studies used Rasch models and none a multidimensional one.

**Methods:**

The present study applies a multidimensional Rasch model using a sample of 518 respondents representative for the Austrian general population aged 18 to 65. A one-dimensional model, a four-dimensional model reflecting the subscale structure suggested by [1], and a four-dimensional model with the background variables gender and age were applied.

**Results:**

While the one-dimensional model showed relatively good fit, the four-dimensional model fitted much better. EAP reliability indices were generally satisfying and the latent correlations varied between 0.31 and 0.88. In the analysis involving background variables, we found a limited effect of the participants’ gender. DIF effects were found unveiling some peculiarities. The two-items subscale Interpersonal Difficulties showed severe weaknesses and the Positive Affect subscale with the reversed item wordings also showed unexpected results.

**Conclusions:**

While a one-dimensional over-all score might still contain helpful information, the differentiation according to the latent dimension is strongly preferable. Altogether, the CES-D can be recommended as a screening instrument, however, some modifications seem indicated.

## Introduction

According to the Global Burden of Disease 2010 study [2], major depressive disorder (MDD) is one of the leading causes for disability with high prevalence causing a substantial economic burden [3]. Although depression is a highly prevalent illness, it is poorly diagnosed in the general health care setting and in non-psychiatric wards [4,5]. Early detection and treatment could reduce impairment in patients, the burden of relatives, and health care costs. Screening instruments facilitate early and correct diagnosis [6] and are essential for epidemiologic studies. Numerous screening tools are available differing in length, psychometric properties, and target population. Wancata et al. [7] discuss crucial attributes a screening instrument must fulfill to be useful for both epidemiologic studies and primary care settings.

The present study focuses on the psychometric properties of the Center of Epidemiologic Studies-Depression Scale [1]. This widely used screening instrument for assessing depressive symptoms frequency in the last week comprises 20 questions. The instrument uses a four-point self-rating response format with the categories 0 = *rarely or none of the time (less than 1 day)*, 1 = s*ome or a little of the time (1–2 days)*, 2 = *occasionally or a moderate amount of time (3–4 days)*, and 3 = *most or all of the time (5–7 days)* allowing for a maximum score of 60. For the score across all items, Radloff (1977)[1] suggested a cut-off value of 16 indicating further clinical evaluation. Based on principal components analysis, she determined four factors from the data comprising the dimensions *Positive Affect* (4 items), *Negative Affect* (7 items), *Somatic Symptoms* (7 items), and *Interpersonal Difficulties* (2 items). Nevertheless, based on the „high internal consistency of the scale found in all groups“, she argued in favour of an overall score to assess „the degree of depressive symptomatology”(p. 398) and against what she considered „undue emphasis on separate factors”(p. 398).

However, from a psychometric point of view, one-dimensionality (i.e., all items cover one and the same latent construct) is a prerequisite for a meaningful interpretation of a total score. Internal consistency alone cannot provide sufficient evidence for the one-dimensionality assumption. Rather, we have to apply more complex and–most importantly–empirically testable models to justify such an assumption. For that purpose, we dispose of either the structural equation modelling family (SEM; [8]) with its special case confirmatory factor analysis (CFA; [9]), or a model from the item response theory family (IRT; also termed Rasch models, RM; [10–12]). Although most of these models were already available in 1977, they were not applied by default at that time and expedient software was not in widespread use.

### Psychometric analyses of the CES-D

Numerous studies have analysed the psychometric properties of the CES-D with special focus on the question of its latent dimensionality. The most basic approach thereby is to apply an exploratory factor analysis/principal component analysis (EFA/PCA; [13]) to determine the required number of latent factors from the data. This strategy has been chosen, for example, by [14–19]. Resulting solutions ranged from 2 to 5 latent factors.

By far more (in fact, most) of the psychometric studies applied a more theory-driven approach by using a confirmatory factor analysis (CFA) in various ways. A combination of EFA and CFA, i.e., exploring and testing, has been applied by [20–27]. These studies applied both the exploratory and the confirmatory factor analysis to the same data sets, thus providing only limited explanatory value regarding the latent dimensionality of the instrument.

A “pure” CFA approach which meansformulating a measurement model on substantive considerations (i.e. a supposed subscale structure expressed, basically, by a factor loading matrix) and testing its adequacy against observed data, has been applied by [28–33]. Several studies employed more complex variants of CFA. These were *(a)* second order CFA (cf. [9]), which assumes a secondary factor behind the (in most cases four) subscale-factors (e.g., [34–43]), *(b)* multi-group-CFA (MG-CFA, cf. [9]), allowing for testing equality constraints across specific sub-samples, such as gender groups (e.g., [44–59]), or *(c)* multiple indicator multiple cause (MIMIC; cf. [9]) or BIFACTOR [60] models, explaining items with more than one latent factor [61–64]. This list makes no claim to be complete, but it demonstrates that we dispose of an impressive body of research regarding the CES-D based on various kinds of factor analyses and SEM approaches.

In contrast, a much smaller number of studies applied IRT models: For example, Stansbury, et al. [65] applied a Rasch model (RM) to a sample of ~2,500 community-dwelling elderly, finding the reverse scored items (4, 8, 12, and 16) not in line with a one-dimensional latent construct and, therefore, eliminated them. But even the reduced set of 16 items still showed deviations from a uni-dimensional construct. Pickard et al. [66] analysed a sample of 101 stroke and 366 primary care patients with the RM, reporting generally good fit except for five items (2, 11, 15, 17, and 19). Gay et al. [67] applied a Rasch analysis to a sample of 347 adults with HIV/AIDS revealing five items (2, 4, 8, 11, and 16) as problematic; however, even their omission would not improve the overall performance of the scale. Kim and Park [68] found in a convenience sample of 183 Korean stroke survivors items 2, 8, and 11 to misfit the RM. Covic et al. [69] and Covic et al. [70] investigated samples of Rheumatoid Arthritis patients with a RM, promoting a 13-items short-version of the CES-D (omitting items 2, 4, 8, 11, 12, 16, and 18) and rescoring the remaining items to a three categorical response format (merging the two middle categories). Two further studies applying an IRT model to the CES-D [71,72] focussed on linking scores of various depression assessments and were therefore not considered in the present article. Table 1 summarizes problematic items identified in the cited studies.

**Table 1:** Items considered problematic in studies applying an IRT model to the CES-D. Bullets indicate items with a significant infit index, bullets and counts in brackets indicate partially problematic items with suspicious thresholds (i.e., significant or outside the critical limits) only.

	good	hopeful	happy	enjoy	blues	depressed	failure	cry	sad	appetite	sleep	unfriendly	dislike	Total
**Subscale**	I	I	I	I	II	II	II	II	II	III	III	IV	IV	
**Item number**	4	8	12	16	3	6	9	17	18	2	11	15	19	
[65]	•	•	•	•										**4**
[66]								•		•	•	•	•	**5**
[67]	•	•		•						•	•			**5**
[68]		•									•			**2**
[69,70]	•	•	•	•					•	•	•			**7**
Present study– 1 dim	•	•	(•)	(•)		•			•		•			**5/(7)**
Present study– 4 dim	•	(•)		(•)	(•)	•	(•)				•			**3/6**
**Total**	**5**	**5/(6)**	**2/(3)**	**3/(5)**	**(1)**	**2**	**(1)**	**1**	**2**	**3**	**6**	**1**	**1**	

### Research question

These results of the IRT analyses indicate that a one-dimensional model seems to not adequately describe the data generating mechanism. The often applied CFA approach allows already for a multidimensional analysis (and the results of these studies support indeed a multi-dimensional structure of the CES-D), however, the CFA model has been originally developed for interval scaled data, assuming linear relationships and a multivariate normal distribution. Although extensions covering ordered categorical data and non-normality exist, the IRT family of models is specifically designed for (ordered) categorical data as we obtain from questionnaires like the CES-D. Amongst others, the IRT approach allows for a detailed analysis of items and item categories, specifically taking into account the categorical response format (for a direct comparison of the various approaches see [73]). To the authors’ knowledge, the CES-D has so far not been analysed with a multidimensional IRT model. Moreover, the present study is the first to also take background variables into account.

Misfit of models applied so far could very well be due to the fact that the CES-D has been applied in specific populations (HIV/AIDS, community-dwelling elderly, stroke & primary care patients, and stroke survivors), although it has originally been designed for “general population surveys” [1] (p. 386]. Hence, the results obtained so far are of limited value, as it remains unclear, whether they also apply to the general population. To shed light on this open question, the present study uses a representative sample from the general population. To the authors’ knowledge, this study is the first one analyzing the CES-D on the basis of a representative sample using a multi-dimensional IRT model.

## Methods

### Sample

The sample consisted of 518 respondents randomly selected from a large Austrian address broker’s data base of phone numbers covering approximately 75% of the Austrian population according to the seller’s information. The sample covered persons aged 18–65 years. Because no population register is available to us, a simple random sample would not be feasible and we applied a complex sampling scheme: Austria has 9 provinces, which have key responsibilities in certain public health issues relevant to our research question. Therefore, we decided to represent them accordingly in the sample by stratification. As the data collection is based on face-to-face interviews, the routes to the households have to be taken into account. Therefore, we used within each stratum a cluster sampling scheme based on districts, which are available in the data base. Based on logistic and financial capabilities, we decided to sample a total of 40 districts, which were drawn at random taking proper shares of urban vs. rural regions into account. The required number of respondents per district was determined proportionally to the respective gender shares and district sizes. The resulting number of male and female respondents per district was drawn at random from the districts addresses in the data base. The sample size has been chosen in line with general recommendation, for example as given by [74], stating that 500 establishes a “Size for most purposes” even under “Adverse Circumstanes” (p. 328).

First, a notification letter informing about study aims and processes was sent to the selected respondents. Then, study workers called each person by phone and asked for permission to visit them for performing the interviews and filling out the questionnaire. Those agreeing to the interview were visited at home. Persons, who were not reached (e.g., due to change of address or phone number) or refused study participation were replaced by further addresses from a back-up list sampled in the same way as the primary list.

### Assessments

Psychiatric case identification was performed by using the SCAN 2.0, the Schedules for Clinical Assessment in Neuropsychiatry [75]. The SCAN is a semi-structured clinical interview designed for use by psychiatrists and clinical psychologists. Every symptom in SCAN is defined in detail [76] and wording is suggested for eliciting each symptom. However, interviewers had to continue inquiring until they dispose of sufficient information to decide whether or not symptom definitions were fulfilled. Its feasibility and reliability have been tested in international field trials [75]. Diagnoses were given according to ICD-10 [77] using a computer algorithm provided for SCAN. Only current disorders (occurring during the 4 weeks before interview) were evaluated in the present study. Eleven psychologists were recruited as interviewers, who were trained by experienced staff from one of the WHO-designated SCAN training centres. All interviewers performed several pilot SCAN interviews before data collection started.

Study participants could decide whether they wanted to start with the questionnaire or the research interview. Either way, interviewers were not aware of the CES-D results. Study participants were included only if they had signed the informed consent. The study was approved by the Ethics Committee of the Medical University of Vienna.

### Model

The response format of the CES-D provides four categories requiring a polytomous version of the Rasch model. One frequently applied model of this kind is the partial credit model (PCM; [78]). However, the PCM is a one-dimensional Rasch Model, i.e., we cannot describe more than one subscale at a time. We also dispose of multidimensional IRT models, which assume more than one latent dimension to generate the responses (cf. [79]). A versatile multidimensional formulation is the multidimensional random coefficients multinomial logit model (MRCMLM; [80]). It covers multidimensionality and allows for controlling for background variables, which each latent factor can be regressed upon. We used a between-item-multidimensional formulation, i.e., each item is associated with exactly one latent factor (cf.[79]). Our analysis strategy was to apply first a one-dimensional model and contrast it to (a) the four-dimensional model and (b) the four-dimensional with background variables. Finally, we performed a differential item functioning analysis (DIF; [81]) to identify potentially problematic items.

For assessing model fit, we use the infit measure [82, 83], the ideal value of which is one. Values larger than one indicate an increasing amount of responses differing from what the model would predict. Values below one indicate responses showing lesser variability than expected critical limits for the infit measure were chosen at 0.7 and 1.3 (cf.[84]). Further, the MRCMLM provides the EAP reliability index (based on Expected A Posteriori parameter estimates, cf. [85,86]) for each latent scale, which can be seen as an equivalent to the classical reliability measure, but for Rasch models; its value should be close to one. For comparing models we use the information based indices AIC [87], the bias corrected AIC (AICc; [88,89]), the bayesian information criterion (BIC; [90]), the adjusted BIC (aBIC; [91]), and the consistent AIC (CAIC; [92]). Information based indices allow for comparing competing models applied to the same data set, with smaller values indicating better over-all model fit. Moreover, we compare nested models with the likelihood ratio test (LRT; [93]).

We used R [94] for all calculations and graphics and the R-package Test Analysis Module (TAM; [95]) for the MRCMLM. A critical alpha of 5% (0.05) was applied for inferential assessment.

## Results

### Sample description

Our sample consisted of 518 participants aged 21 to 67 years (M = 46.6, SD = 13.3); 264 (51%) of them were female. Regarding education, 238 (46,1%) had a university entrance diploma (termed “Matura” in Austria) and 24 (4.6%) were still in education. Thirty-six respondents (6.9%) declared to be unemployed while 364 (70.3%) were employed.

### The one-dimensional model

First, a one-dimensional Rasch model for polytomous data (i.e., a PCM) was applied. This model constitutes the reference model, against which the more complex approaches will be tested. The EAP reliability index of the latent scale of this model was 0.795.

**Fig 1:**
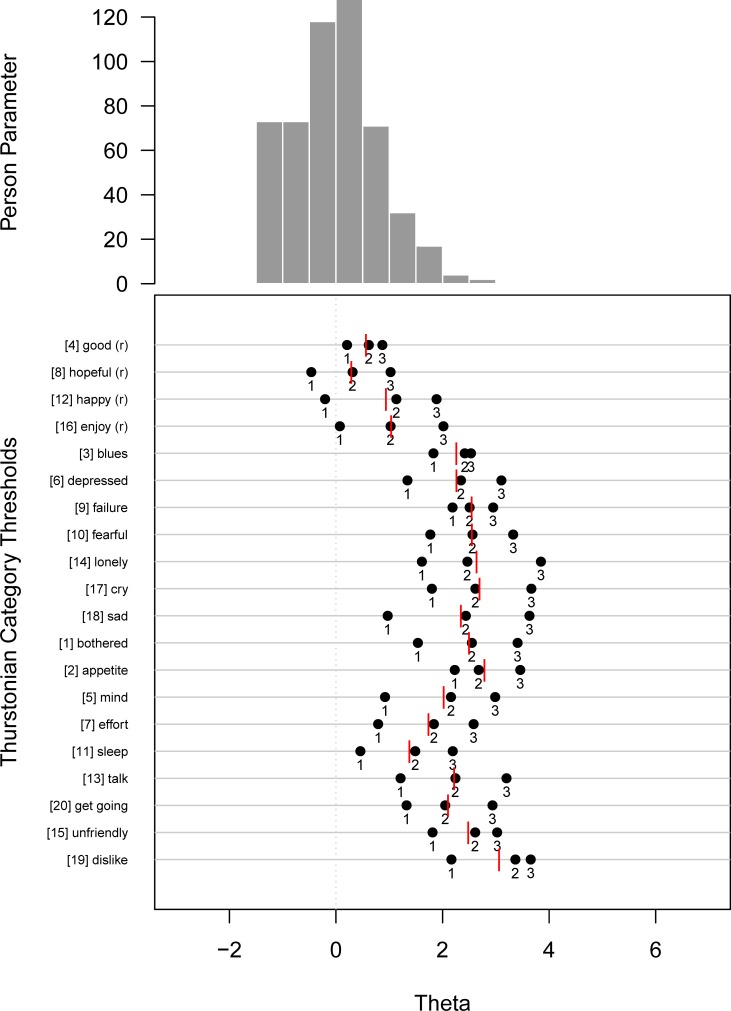
Person-item map of the one-dimensional model. The upper part shows the histogram of the person parameter distribution and the lower plot the location of the Thurstonian thresholds, both sharing the same metric. The red lines in the lower diagram indicate the average threshold of each item, constituting a measure of the “difficulty”of this item. Items are sorted according to subscales as indicated by Radloff (1977).

Fig 1 shows the person-item-map ([83]; a detailed treatment give [96]) of the one-dimensional model. The horizontal axis denotes the latent dimension representing “over-all”-depression (in contrast to the specific depression facets in the next model). From the histogram in the upper part we learn that the majority of the sample exhibits low depression values. In contrast, we find the majority of the thresholds in the higher regions of this latent dimension, indicating that only respondents with higher depression values are likely to choose the according response categories. Especially for items 2 (appetite), 9 (failure), 10 (fearful), 15 (unfriendly), and 19 (dislike), even the threshold between categories 0 and 1 is located considerably high. This means that these items are “difficult”from a psychometric point of view thus requiring a higher latent score to endorse them. Accordingly, the thresholds of the subscale I (*Positive Affect*), i.e., items 4 (good), 8 (hopeful), 12 (happy), and 16 (enjoy), are located in the lower regions of the latent dimension. One peculiarity becomes evident: The thresholds of items 3 (blues), 4 (good), and 9 (failure) are considerably close to each other indicating that these items do not differentiate very much across the latent dimension.

**Fig 2:**
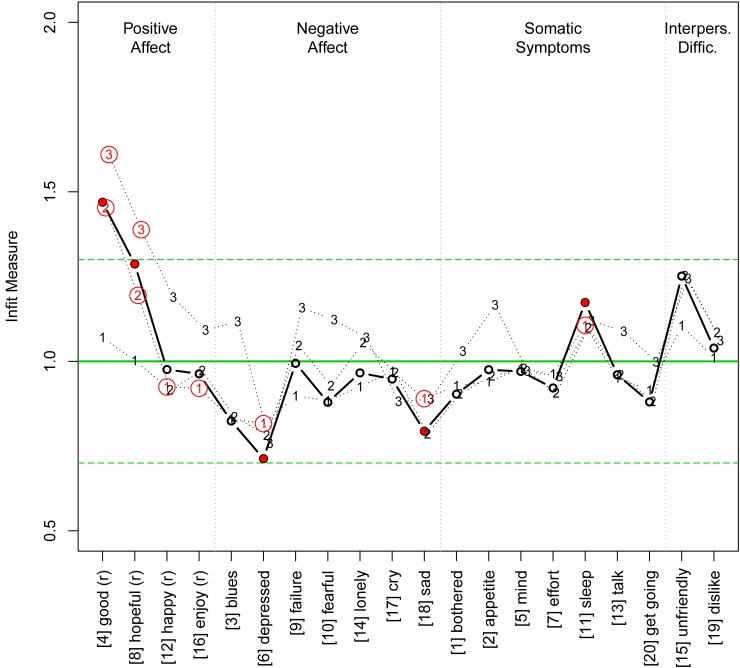
Infit measures of the one-dimensional model. Notes: The bold line shows the item infit with bullets indicating significant values. The dotted lines indicate the infit values of the three thresholds (labelled with 1, 2, and 3; slightly horizontally displaced for better readability).The bold horizontal line indicates the ideal value of 1 and the two dashed horizontal lines the limits of acceptability (0.7 to 1.3). Numbers in circles indicate significant thresholds (note that the significance also depends on the standard error of the respective estimate, hence, significant values need not be located outside the acceptability limits and similar values need not be significant at the same time). The (r) indicates that the item codings had to be reversed prior to evaluation, because these items were positively worded. The items along the horizontal axis are sorted according to the four subscales with dotted vertical lines showing the subscale blocks with their original number in brackets.

Fig 2 shows the infit measures and the thresholds of the 20 CES-D items. Most of the values appear in the vicinity of 1, hence, the global impression is good. However, some items show peculiarities: The four items of subscale I show elevated item infit with statistically significantly deviating thresholds; thresholds 2 and 3 of the items 4 (good) and 8 (hopeful) are significant and three of them also lie above the upper limit of 1.3; further, thresholds 1 of items 12 (happy) and 18 (enjoy) are below the ideal value of 1 and were significant. In subscale II, item 6 (depressed) was close to the lower limit and significant; its first threshold was significant as well. The same applies to item 18 (sad). Finally, in subscale III, item 11 (sleep) was larger than 1 and significant.

### The four-dimensional model

Next, we applied a four-dimensional model according to the item allocation as proposed by [1]. The EAP reliability indices for the 4 latent dimensions were 0.699 for *Positive Affect* (henceforth termed subscale I), 0.730 for *Negative Affect* (subscale II), 0.727 for *Somatic Symptoms* (III), and 0.451 for *Interpersonal Difficulties* (IV). Table 2 lists the information based indices indicating that the four-dimensional model describes the data better than the one-dimensional model.

**Table 2:** Information based fit indices for the one- and the four-dimenensional model.

	1-Dim	4-Dim
**log Lik**	–6464.67	–6450.43
**num Par**	70	78
**AIC**	13450	13069
**AICc**	13467	13092
**BIC**	13709	13366
**aBIC**	13515	13144
**CAIC**	13770	13436

**Fig 3:**
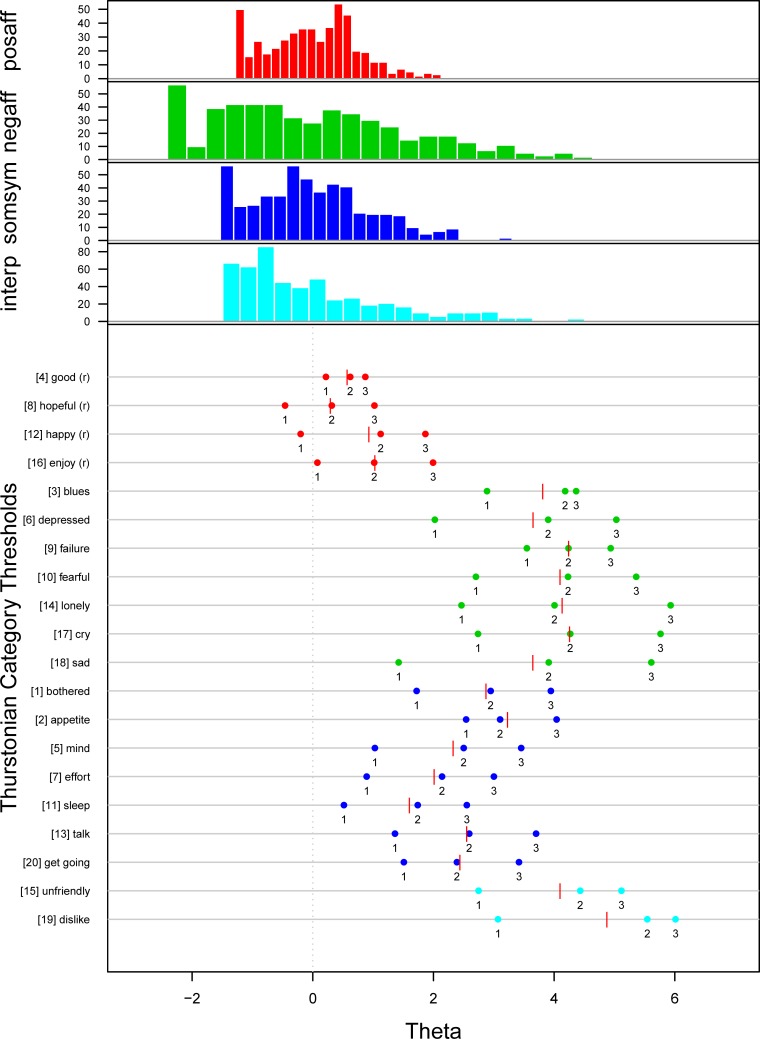
Person-item-map of the 4-dimensional model. The upper part of the plot shows the histogram of the person parameter estimates for each of the four subscales. The colors indicate the subscales. For further notes see Fig 1.

Also, the direct model comparison via the likelihood ratio test (LRT) identified the four-dimensional model to fit the data significantly better than the one-dimensional one (*χ*^2^ = 398.77; *df* = 9; *p <* 1e–10). Fig 3 shows the person-item-map of the four-dimensional model.

The histogram of the person parameter estimates shows again that most respondents exhibit low values of depression, with subscale II (*Negative Affect*) covering a wider range than the other three subscales. The item category thresholds show a similar pattern as in the one-dimensional case. However, the thresholds of the four-dimensional model cover a much broader range of values. Nevertheless, items 4 (good) and 8 (hopeful) still show thresholds considerably close to each other, which means that these two items still do not discriminate very well across the spectrum of depression, i.e. respondents chose predominantly either category 0 (not at all) or category 3 (all the time).

**Fig 4:**
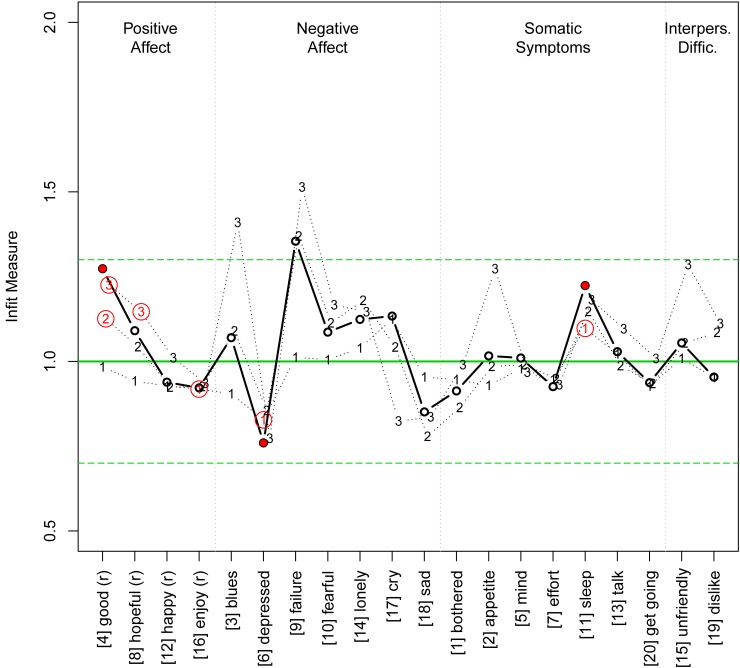
Infit measures of the four-dimensional model. For notes see Fig 2.

Fig 4 shows the infit indices for the 20 CES-D items. Again, we find a few peculiarities in scale I, yet to a lesser degree: The thresholds 2 and 3 of item 4 (good) and threshold 3 of item 8 (hopeful) are still significant, but the infit measure is below the critical limit of 1.3. Interestingly, now the items 3 (blues), 9 (failure), and 10 (fearful) show infit measures above the critical limit of 1.3. Again, item 6 (depressed) and item 11 (sleep) have thresholds deviating significantly from the ideal value of 1.

Table 3 shows the correlation matrix of the four latent dimensions (main diagonal entries denote the variances of each latent dimension).

**Table 3:** Correlations of the latent subscales. Note: The entries in the main diagonal (italicized) are the variance of each subscale.

	Pos.Aff.	Neg.Aff.	Som.Symp.	Interpers.Diff.
**Pos.Aff.**	*0*.*911*			
**Neg.Aff.**	0.554	*1*.*912*		
**Som.Symp.**	0.586	0.875	*1*.*165*	
**Interpers.Diff.**	0.310	0.595	0.477	*1*.*905*

The highest correlation was found between *Negative Affect* and *Somatic Symptoms* (.88) while the weakest correlation occurred between *Positive Affect* and *Interpersonal Difficulties* (.31); the remaining correlation coefficients were mediocre (between 0.48 and 0.59).

### The four-dimensional model with background variables

Finally, the multidimensional model has been extended by the two background variables gender and age. Regarding model-fit, we find the person-item-map almost identical to that of the four-dimensional model without background variables (therefore not presented here; the same applies to the infit plot; interested readers can request a copy of these plots from the authors). The EAP reliability indices for the four latent dimensions were marginally better than for the previous model (I: 0.702; II: 0.740; III: 0.730; IV: 0.455). A direct model comparison using information based indices or the LRT is not possible, because this model was applied to a different data set (with the two background variables added).

The most interesting results of this model are the regression coefficients of the two background variables upon the four latent dimensions (see Table 4).

**Table 4:** Regression coefficients of the latent background model.

	Pos. Aff.	Neg. Aff.	Som. Symp.	Int.Diff.
**Intercept**	0	0	0	0
**Gender**	-0,11325	0,72233	0,24627	0,43946
**Age**	0,00445	-0,00421	-0,00986	-0,00365

Regarding the impact of gender upon the subscales, we find two effects for the latent dimensions *Negative Affect* and *Interpersonal Difficulties*. In contrast, the respondents’ age did not reveal any notable influence. From these results, we learn that gender but not age seems to play a role for the CES-D. This will be pursued further in the following DIF-analysis, which delivers more detailed insights.

### DIF analysis

We split the sample according to gender on the one hand and a diagnosis of depression within the last month as split criteria for the DIF analysis–the former, because it proved to be influential as background variable, and the latter, because the CES-D has been developed to measure depression in the general population. Therefore, it is of particular interest, if there are items operating different in depressed people than in non-depressed-ones. We used the four-dimensional model without background variables for the DIF analyses, because controlling for gender or depression would eliminate possible effects we are looking for in this analysis step.

**Fig 5:**
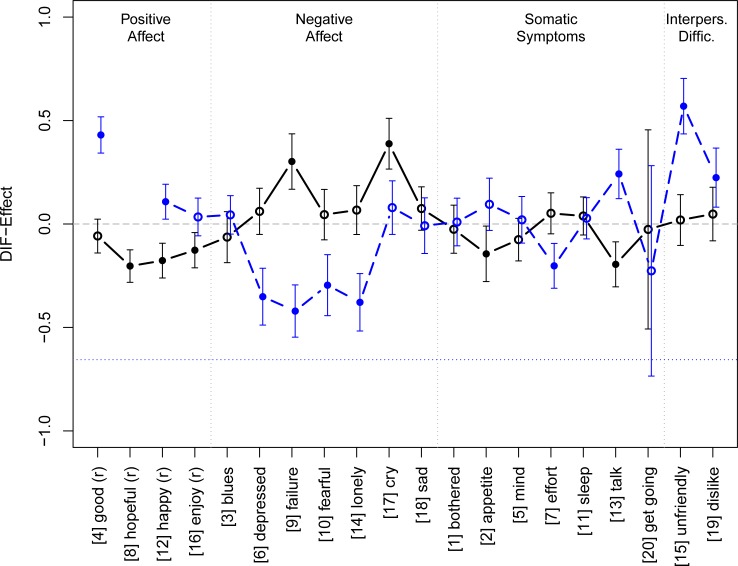
Differential item functioning due to gender and depression. Notes: The dots represent the DIF-Effect, i.e., the item parameter difference between the two groups; error bars indicate the 95% confidence interval; Bullets indicate a significant DIF-Effect for the respective item. Values below 0 means that the item is rather preferred by men, items with DIF-values above 0 are rather preferred by women. The solid line indicates DIF according to sex and the dashed line indicates DIF according to depression. In the latter case, item 2 had to be omitted due to technical reasons (see text). For better readability, the two curves were horizontally displaced.

First, we will focus upon the global DIF-effect. Here, we find a weak general DIF-effect for gender (global effect parameter –0.103; 95% CI = -0.13/-0.07), i.e., women were slightly (but significantly) more likely to endorse all items. Because such an over-all effect is little informative, we turn to an item-wise analysis. Fig 5 presents the item-wise DIF-effects according to gender (solid line).

Seven items show a significant yet moderate DIF effect. The *Positive Affect* subscale is affected the most with three out of four items (hopeful, happy, enjoy) showing DIF in favour of men (i.e., men are more likely to endorse these items than women). There is a DIF-effect in favour of women for two of the *Negative Affect* subscale items (failure, cry) and in favour of men for two items of the *Somatic Symptoms* subscale (appetite, talk).

For the second DIF analysis, we split the sample into respondents with vs. without a diagnosis of depression according to SCAN. Other diagnoses were excluded for this step, resulting in a slight sample reduction (*n*_*red*_ = 452). Item 2 (appetite) had to be excluded from the analysis for technical reasons (response category 3 did not occur in the reduced sample). There was a global effect with depressed respondents more likely endorsing all items. (effect parameter -0.656; 95% CI = –0.62/–0.69). Fig 5 shows the item-wise DIF-effects (dashed line). We find significant effects for 10 items: For depressed respondents, it was more difficult to endorse items 4 (good), 8 (hopeful), 13 (talk), 15 (unfriendly), and 19 (dislike) and more easy to endorse items 3 (blues), 6 (depressed), 9 (failure), 10 (fearful), and 7 (effort). Although most of these effects were statistically significant, they can be considered small from a substantive perspective. The largest effect was observed for items 15 (unfriendly), 19 (dislike), and 13 (talk), which were more difficult to endorse for respondents fulfilling depression criteria.

## Discussion

The present study analysed the CES-D with a multi-dimensional IRT model in a sample representative for the general population. A one-dimensional solution was contrasted to a four-dimensional model reflecting the subscales as asserted by [1]. Interestingly, the fit of the one-dimensional model was already considerably good. Only item 1 (bothered) showed an infit value outside the usual limits of acceptability, and a few thresholds of the remaining items reached statistical significance. The EAP reliability measure of this model was 0.8, which can be regarded as fairly satisfying. Hence, we can conclude that an overall-score would deliver quite useful information. This finding supports the view of Radloff [1] advocating the use of the total score of the CES-D, however, based on a much more elaborated methodological foundation. This could be advantageous, for example, when using the CES-D as a screening instrument in a multistep diagnostic process, where a single total score with a certain cut-off value would be preferable.

However, the fit of the four-dimensional model was by far (and significantly) better than the fit of the one-dimensional model. It is also in line with the meta study of Shafer (2006), who also found “strongest support (…) for the four-factor structure of the CES-D” [97] (p. 136). The reliability coefficients of the subscales revealed that subscales I, *Positive Affect*, II, *Negative Affect*, and III, *Somatic Symptoms* achieve values in the vicinity of 0.7, which is satisfying, while subscale IV, *Interpersonal Difficulties* was mediocre at best (0.45). When comparing reliability indices of the four- and the one-dimensional model, we have to keep in mind that reliability depends–amongst other things–on scale length as well. In the one-dimensional model, a common scale is built from all 20 items, while the subscales of the four-dimensional model are much shorter, therefore, the subscale indices are lower for technical reasons. Taking this into account, we consider the reliability indices of the subscales I-III as sufficiently high. The poor result of subscale IV implies that two items would not suffice to establish a meaningful subscale. Such short scales are rather useful for screenings in the first step of a two-step screening procedure fostering a decision regarding further diagnostic procedures (cf. [98–100]). However, they are hardly suitable for the quantitative assessment of a trait. In the present case, *Interpersonal Difficulties*–which is a rather complex construct–would be measured with a score consisting of two items and a total value ranging from 0 to 6. Hence, the interpretation of this scale is very limited and should be done with great caution (if at all).

Comparing the present results to those of the previously reported IRT-based studies, we find largely agreeing and some interesting new results: Generally, the one-dimensional model rendered seven items suspicious (five with significant infit plus two with significant thresholds only), whereas the four-dimensional model only showed significant infit for three items and suspicious thresholds for another 3 items. This is in line with the previous results, again showing the four-dimensional model to be superior to the one-dimensional model. We will, therefore, focus on this model in the discussion of item fit: Regarding subscale I, *Positive Affect*, item 4 (good) proved most problematic, as not only was its infit measure significant, but also thresholds 2 and 3. Items 8 (hopeful) and 16 (enjoy) had one problematic threshold each. Interestingly, item 12 (happy) worked well here, in contrast to [66] and [70, 71]. For subscale II, *Negative Affect*, we find diverging results, as the suspicious items 3 (blues), 6 (depressed), and 9 (failure) have not been reported problematic in the previous studies. Taking into consideration that these items cover core symptoms of depression, our results might reflect the different populations in which the CES-D was used. Our study covered the general population, where these statements may play a different role compared to the specific populations reported in the previous studies. The DIF analysis discussed below will shed further light on this issue. For subscale III, *Somatic Symptoms*, the situation is fairly clear: Item 11 (sleep) was suspicious, which is in line with four out of the five reported studies. In contrast, item 2 (appetite) was inconspicuous in contrast to [67,68,70,71]. Interestingly, the infit measures of the two items of subscale IV, *Interpersonal Difficulties*, were satisfying in our study. Further details regarding the results and the discussion of our analyses can be found in the online supplemental material S1 File.

As a limitation, we have to take into account that the sample relies on a phone number data base, which will not cover the entire population of a country. Therefore, slight peculiarities may still exist. However, we consider this limitation tolerable for two reasons: First, it is unlikely that our results are severely biased as the data base still covers an enormous portion of the entire population. Second, Rasch models are “sample independent” [101], which, in short, describes the fact that item parameter estimates do not depend on the person parameter distribution and vice versa [102,103]. We therefore regard our results as dependable.

Concluding, we can state that the one-dimensional modelling approach proved clearly inferior to the multidimensional one. This is in line with previous studies: For example, Gay et al. [67] also used the PCM approach and found violations of the one-dimensionality assumption for all 20 items of the CES-D. Moreover, we found subscale IV, *Interpersonal Difficulties*, to exhibit severe limitations from a psychometric point of view. Therefore, it should be handled with care. Apart from that and a few limitations deserving further elaboration, analyses of the subscales yielded convincing results supporting the subscale structure of the CES-D. Therefore, although not entirely dismissing the overall score, we advocate the use of a subscale based interpretation due to its superior psychometric qualities.

## Supporting information

S1 File Supplemental discussion. (PDF)

S2 File Data. (CSV)

## References

[1] Radloff LS. The CES-D scale: A self-report depression scale for research in the general population. Appl Psychol Meas. 1977;1(3):385–401.

[2] Vos T, Flaxman AD, Naghavi M, Lozano R, Michaud C, Ezzati M, et al Years lived with disability (YLDs) for 1160 sequelae of 289 diseases and injuries 1990–2010: a systematic analysis for the Global Burden of Disease Study 2010. Lancet. 2012;380(9859):2163–2196. doi: 10.1016/S0140-6736(12)61729-2 23245607 10.1016/S0140-6736(12)61729-2 PMC6350784

[3] Luppa M, Heinrich S, Angermeyer MC, König HH, Riedel-Heller SG. Cost-of-illness studies of depression: a systematic review. J Affect Dis. 2007;98(1):29–43. 16952399 10.1016/j.jad.2006.07.017

[4] Wancata J, Windhaber J, Bach M, Meise U. Recognition of psychiatric disorders in nonpsychiatric hospital wards. J Psychosom Res. 2000;48(2):149–155. 10719131 10.1016/s0022-3999(99)00098-7

[5] Üstün TB, Sartorius N. Mental illness in general health care: an international study New York: Wiley; 1995.

[6] U.S. preventive service task force. Screening for Depression: Recommendations and Rationale. Ann Int Med. 2002;36:760–764. 10.7326/0003-4819-136-10-200205210-00012 12020145

[7] Wancata J, Marquart B, Weiss M, Krautgartner M, Friedrich F, Alexandrowicz R. [Screening Instruments for Depression]. Psychosomatik und Konsiliarpsychiatrie. 2007;1(2):144–153.

[8] Bollen KA. Structural Equations with Latent Variables. New York: Wiley & Sons; 1989.

[9] Brown TA. Confirmatory factor analysis for applied research New York: Guilford; 2006.

[10] de Ayala RJ. The theory and practice of item response theory New York: Guilford; 2013.

[11] Embretson SE, Reise SP. Item response theory for psychologists Mahwah: Lawrence Erlbaum; 2000.

[12] van der Linden WJ, Hambleton RK, editors. Handbook of modern item response theory. New York: Springer; 1997.

[13] Mulaik SA. Foundations of factor analysis 2nd ed. Boca Raton: CRC Press; 2009.

[14] Amer MM, Awad GH, Hovey JD. Evaluation of the CES-D Scale factor structure in a sample of second-generation Arab-Americans. Int J Cult Ment Health. 2014;7(1):46–58.

[15] Devins GM, Orme CM, Costello CG, Binik YM, Frizzell B, Stam HJ, et al Measuring depressive symptoms in illness populations: Psychometric properties of the Center for Epidemiologic Studies Depression (CES-D) scale. Psychol Health. 1988;2(2):139–156.

[16] Lacasse JJ, Forgeard MJ, Jayawickreme N, Jayawickreme E. The factor structure of the CES-D in a sample of Rwandan genocide survivors. Soc Psychiatry Psychiatr Epidemiol. 2014;49(3):459–465. doi: 10.1007/s00127-013-0766-z 24173407 10.1007/s00127-013-0766-z

[17] Leykin Y, Torres LD, Aguilera A, Muñoz RF. Factor structure of the CES-D in a sample of Spanish- and English-speaking smokers on the Internet. Psychiatry Res. 2011;185(1):269–274. 20684849 10.1016/j.psychres.2010.04.056 PMC4165607

[18] Schroevers MJ, Sanderman R, van Sonderen E, Ranchor AV. The Evaluation of the Center for Epidemiologic Studies Depression (CES-D) Scale: Depressed and Positive Affect in Cancer Patients and Healthy Reference Subjects. Qual Life Res. 2000;9:1015–1029. 11332223 10.1023/a:1016673003237

[19] Roberts RE, Vernon SW, Rhoades HM. Effects of language and ethnic status on reliability and validity of the Center for Epidemiologic Studies-Depression Scale with psychiatric patients. J Nerv Ment Dis. 1989;177(10):581–592. 2677237 10.1097/00005053-198910000-00001

[20] Asakura T, Gee GC, Asakura K. Assessing a culturally appropriate factor structure of the Center for Epidemiologic Studies Depression (CES-D) scale among Japanese Brazilians. Int J Cult Ment Health. 2015;8(4):426–445.

[21] Edman JL, Danko GP, Andrade N, McArdle JJ, Foster J, Glipa J. Factor structure of the CES-D (Center for Epidemiologic Studies Depression scale) among Filipino-American adolescents. Soc Psychiatry Psychiatr Epidemiol. 1999;34(4):211–215. 10365627 10.1007/s001270050135

[22] Helmes E, Nielson WR. An examination of the internal structure of the Center for Epidemiological Studies-Depression Scale in two medical samples. Pers Individ Dif. 1998;25(4):735–743.

[23] Kim JH, Park EY. The factor structure of the center for epidemiologic studies depression scale in stroke patients. Top Stroke Rehabil. 2012;19(1):54–62. doi: 10.1310/tsr1901-54 22306629 10.1310/tsr1901-54

[24] Losada A, de los Ángeles Villareal M, Nuevo R, Márquez-González M, Salazar BC, Romero-Moreno R, et al Cross-cultural confirmatory factor analysis of the CES-D in Spanish and Mexican dementia caregivers. Span J Psychol. 2012;15(02):783–792. 22774452 10.5209/rev_sjop.2012.v15.n2.38890

[25] O'Rourke N. Factor structure of the Center for Epidemiologic Studies–Depression Scale (CES–D) among older men and women who provide care to persons with dementia. Int J Testing. 2005;5(3):265–277.

[26] Privado J, Garrido J. Factorial structure of the Spanish center for epidemiologic studies depression scales in HIV patients. Community Ment Health J. 2013;49(4):492–497. doi: 10.1007/s10597-013-9618-2 23756721 10.1007/s10597-013-9618-2

[27] Teraoka M, Kyougoku M. Analysis of structural relationship among the occupational dysfunction on the psychological problem in healthcare workers: a study using structural equation modeling. PeerJ. 2015;3:e1389 doi: 10.7717/peerj.1389 26618078 10.7717/peerj.1389 PMC4655103

[28] Brown J, Jose P, Ng SH, Guo J. Psychometric properties of three scales of depression and well-being in a mature New Zealand sample. NZ J Psychol. 2002;31(1):39.

[29] Dick RW, Beals J, Keane EM, Manson SM. Factorial structure of the CES-D among American Indian adolescents. J Adolesc. 1994;17(1):73–79.

[30] Edwards MC, Cheavens JS, Heiy JE, Cukrowicz KC. A reexamination of the factor structure of the Center for Epidemiologic Studies Depression Scale: is a one-factor model plausible?. Psychol Assess. 2010;22(3):711 doi: 10.1037/a0019917 20822284 10.1037/a0019917 PMC3660842

[31] Johnson CS, Mcleod PJ, Sharpe D, Johnston EM. Differences among core dimensions of the Centre for Epidemiological Studies Depression (CES-D) scale across age and gender groups. Can J Commun Ment Health. 2008;27(1):79–91.

[32] McCauley SR, Pedroza C, Brown SA, Boake C, Levin HS, Goodman HS, Merritt SG. Confirmatory factor structure of the Center for Epidemiologic Studies-Depression scale (CES-D) in mild-to-moderate traumatic brain injury. Brain Inj. 2006;20(5):519–527. doi: 10.1080/02699050600676651 16716998 10.1080/02699050600676651

[33] Rozario PA, Menon N. An examination of the measurement adequacy of the CES-D among African American women family caregivers. Psychiatr Res. 2010;179(1):107–112. 10.1016/j.psychres.2010.06.022 PMC2921877 20663570

[34] Boisvert JA, McCreary DR, Wright KD, Asmundson GJ. Factorial validity of the center for epidemiologic studies‐depression (CES‐D) scale in military peacekeepers. Depress Anxiety. 2003;17(1):19–25. doi: 10.1002/da.10080 12577274 10.1002/da.10080

[35] Cheng CP, Yen CF, Ko CH, Yen JY. Factor structure of the center for epidemiologic studies depression scale in Taiwanese adolescents. Compr Psychiatry. 2012;53(3):299–307. doi: 10.1016/j.comppsych.2011.04.056 21621755 10.1016/j.comppsych.2011.04.056

[36] Davidson H, Feldman PH, Crawford S. Measuring depressive symptoms in the frail elderly. J Gerontol. 1994;49(4):P159–P164. 8014396 10.1093/geronj/49.4.p159

[37] Lee SW, Stewart SM, Byrne BM, Wong JP, Ho SY, Lee PW, et al Factor structure of the Center for Epidemiological Studies Depression scale in Hong Kong adolescents. J Pers Assess. 2008;90(2):175–184. doi: 10.1080/00223890701845385 18444112 10.1080/00223890701845385

[38] McCallum J, Mackinnon A, Simons L, Simons J. Measurement Properties of the Center for Epidemiological Studies Depression Scale: an Australian Community Study of Aged Persons. J Gerontol. 1995;50B:182–189. 10.1093/geronb/50b.3.s182 7767702

[39] Mogos MF, Beckstead JW, Kip KE, Evans ME, Boothroyd RA, Aiyer AN, et al Assessing Longitudinal Invariance of the Center for Epidemiologic Studies-Depression Scale Among Middle-Aged and Older Adults. J Nurs Meas. 2015;23(2):302–314. doi: 10.1891/1061-3749.23.2.302 26284842 10.1891/1061-3749.23.2.302

[40] Phillips GA, Shadish WR, Murray DM, Kubik M, Lytle LA, Birnbaum AS. The center for epidemiologic studies depression scale with a young adolescent population: A confirmatory factor analysis. Multivariate Behav Res. 2006;41(2):147–163. doi: 10.1207/s15327906mbr4102_3 26782908 10.1207/s15327906mbr4102_3

[41] Rhee SH, Petroski GF, Parker JC, Smarr KL, Wright GE, Multon KD, et al A confirmatory factor analysis of the Center for Epidemiologic Studies Depression Scale in rheumatoid arthritis patients: additional evidence for a four-factor model. Arthritis Care Res. 1999;12(6):392–400. 11081010 10.1002/1529-0131(199912)12:6<392::aid-art7>3.0.co;2-9

[42] Ros L, Latorre JM, Aguilar MJ, Serrano JP, Navarro B, Ricarte JJ. (2011). Factor structure and psychometric properties of the center for epidemiologic studies depression scale (CES-D) in older populations with and without cognitive impairment. Int J Aging Hum Dev. 2011;72(2):83–110. doi: 10.2190/AG.72.2.a 21639012 10.2190/AG.72.2.a

[43] Sheehan TJ, Fifield J, Reisine S, Tennen H. The measurement structure of the Center for Epidemiologic Studies Depression scale. J Pers Assess. 1995;64(3):507–521. doi: 10.1207/s15327752jpa6403_9 7760258 10.1207/s15327752jpa6403_9

[44] Assari S, Moazen-Zadeh E. Confirmatory Factor analysis of the 12-item center for epidemiologic studies Depression scale among Blacks and Whites. Front Psychiatry. 2016;7:178 doi: 10.3389/fpsyt.2016.00178 27872599 10.3389/fpsyt.2016.00178 PMC5098257

[45] Breithaupt K, Zumbo BD. Sample invariance of the structural equation model and the item response model: a case study. Struct Equ Modeling. 2002;9(3):390–412.

[46] Chen H, Mui AC. Factorial validity of the Center for Epidemiologic Studies Depression Scale short form in older population in China. Int Psychogeriatr. 2014;26(01):49–57. 24125553 10.1017/S1041610213001701

[47] Coman EN, Lordache E, Schensul JJ, Coiculescu I. Comparisons of CES‐D depression scoring methods in two older adults ethnic groups. The emergence of an ethnic‐specific brief three‐item CES‐D scale. Int J Geriatr Psychiatry, 2013;28(4):424–432. doi: 10.1002/gps.3842 22674637 10.1002/gps.3842

[48] Ferro MA, Speechley KN. Factor structure and longitudinal invariance of the Center for Epidemiological Studies Depression Scale (CES-D) in adult women: application in a population-based sample of mothers of children with epilepsy. Arch Womens Ment Health. 2013;16(2):159–166. doi: 10.1007/s00737-013-0331-5 23420273 10.1007/s00737-013-0331-5

[49] Gomez R, McLaren S. The Center for Epidemiologic Studies Depression Scale: Invariance across heterosexual men, heterosexual women, gay men, and lesbians. Psychol Assess. 2017;29(4):361 doi: 10.1037/pas0000352 27362464 10.1037/pas0000352

[50] Liang J, Tran TV, Krause N, Markides KS. Generational differences in the structure of the CES-D scale in Mexican Americans. J Gerontol. 1989;44(3):S110–S120. 2715592 10.1093/geronj/44.3.s110

[51] Makambi KH, Williams CD, Taylor TR, Rosenberg L, Adams-Campbell LL. An assessment of the CES-D scale factor structure in black women: The Black Women’s Health Study. Psychiatry Res. 2009;168:163–170. doi: 10.1016/j.psychres.2008.04.022 19501414 10.1016/j.psychres.2008.04.022 PMC2704501

[52] McArdle JJ, Johnson RC, Hishinuma ES, Miyamoto RH, Andrade NN. Structural equation modeling of group differences in CES-D ratings of native Hawaiian and non-Hawaiian high school students. J Adolesc Res, 2001;16(2):108–149.

[53] Missinne S, Vandeviver C, Van de Velde S, Bracke P.Measurement equivalence of the CES-D 8 depression-scale among the ageing population in eleven European countries. Soc Sci Res. 2014;46:38–47. doi: 10.1016/j.ssresearch.2014.02.006 24767588 10.1016/j.ssresearch.2014.02.006

[54] Posner SF, Stewart AL, Marín G, Pérez-Stable EJ. Factor variability of the center for epidemiological studies depression scale (CES-D) among urban latinos. Ethn Health. 2001;6(2):137–144. doi: 10.1080/13557850120068469 11488294 10.1080/13557850120068469

[55] Roth DL, Ackerman ML, Okonkwo OC, Burgio LD. The four-factor model of depressive symptoms in dementia caregivers: A structural equation model of ethnic differences. Psychol Aging. 2008;23(3):567 doi: 10.1037/a0013287 18808246 10.1037/a0013287 PMC2579269

[56] Verhoeven M, Sawyer MG, Spence SH. The factorial invariance of the CES-D during adolescence: Are symptom profiles for depression stable across gender and time?. J Adolesc. 2013;36(1):181–190. doi: 10.1016/j.adolescence.2012.10.007 23206757 10.1016/j.adolescence.2012.10.007

[57] Wang M, Armour C, Wu Y, Ren F, Zhu X, Yao S. Factor structure of the CES‐D and measurement invariance across gender in mainland Chinese adolescents. J Clin Psychol. 2013;69(9):966–979. doi: 10.1002/jclp.21978 23775279 10.1002/jclp.21978

[58] Williams CD, Taylor TR, Makambi K, Harrell J, Palmer JR, Rosenberg L, et al CES-D four-factor structure is confirmed, but not invariant, in a large cohort of African American women. Psychiatry Res. 2007;150(2):173–180. doi: 10.1016/j.psychres.2006.02.007 17291596 10.1016/j.psychres.2006.02.007

[59] Yu SC, Lin YH, Hsu WH. Applying structural equation modeling to report psychometric properties of Chinese version 10-item CES-D depression scale. Qual Quant. 2013;47(3):1511–1518.

[60] Holzinger KJ, Swineford F. The bi-factor method. Psychometrika. 1937;2(1):41–54.

[61] Fong TC, Chan CL, Ho RT, Chan JS, Chan CH, Ng SM. Dimensionality of the Center for Epidemiologic Studies Depression Scale: an exploratory bi-factor analytic study. Qual Life Res. 2016;25(3):731–737. doi: 10.1007/s11136-015-1105-5 26282007 10.1007/s11136-015-1105-5 PMC4759208

[62] Gomez R, McLaren S. The center for epidemiologic studies depression scale: support for a bifactor model with a dominant general factor and a specific factor for positive affect. Assessment. 2015;22(3):351–360. doi: 10.1177/1073191114545357 25085880 10.1177/1073191114545357

[63] Grayson DA, Mackinnon A, Jorm AF, Creasey H, Broe GA. Item bias in the center for epidemiologic studies depression scale effects of physical disorders and disability in an elderly community sample. J Gerontol B Psychol Sci Soc Sci. 2000;55(5):P273–P282. 10985292 10.1093/geronb/55.5.p273

[64] Miller TQ, Markides KS, Black SA. The factor structure of the CES-D in two surveys of elderly Mexican Americans. J Gerontol B Psychol Sci Soc Sci. 1997;52(5):S259–S269. 9310098 10.1093/geronb/52b.5.s259

[65] Stansbury JP, Ried LD, Velozo CA. Unidimensionality and bandwidth in the Center for Epidemiologic Studies Depression (CES–D) scale. J Pers Assess. 2006;86(1):10–22. doi: 10.1207/s15327752jpa8601_03 16436016 10.1207/s15327752jpa8601_03

[66] Pickard AS, Dalal MR, Bushnell DM. A comparison of depressive symptoms in stroke and primary care: applying Rasch models to evaluate the center for epidemiologic studies-depression scale. Value Health. 2006;9(1):59–64. doi: 10.1111/j.1524-4733.2006.00082.x 16441526 10.1111/j.1524-4733.2006.00082.x

[67] Gay CL, Kottorp A, Lerdal A, Lee KA. Psychometric limitations of the Center for Epidemiologic Studies-Depression Scale for assessing depressive symptoms among adults with HIV/AIDS: a Rasch analysis. Depress Res Treat. 2016:2824595 doi: 10.1155/2016/2824595 27042347 10.1155/2016/2824595 PMC4794594

[68] Kim JH, Park EY. Rasch analysis of the Center for Epidemiologic Studies Depression scale used for the assessment of community-residing patients with stroke. Disabil Rehabil. 2011;33(21–22):2075–2083. doi: 10.3109/09638288.2011.560333 21401334 10.3109/09638288.2011.560333

[69] Covic T, Pallant JF, Conaghan PG, Tennant A. A longitudinal evaluation of the Center for Epidemiologic Studies-Depression scale (CES-D) in a rheumatoid arthritis population using Rasch analysis. Health Qual Life Outcomes. 2007;5(1):41. 17629902 10.1186/1477-7525-5-41 PMC1950699

[70] Covic T, Pallant JF, Tennant A, Cox S, Emery P, Conaghan PG. Variability in depression prevalence in early rheumatoid arthritis: a comparison of the CES-D and HAD-D Scales. BMC Musculoskelet Disord. 2009;10(1):18. 19200388 10.1186/1471-2474-10-18 PMC2649031

[71] Lambert SD, Clover K, Pallant JF, Britton B, King MT, Mitchell AJ, et al Making Sense of Variations in Prevalence Estimates of Depression in Cancer: A Co-Calibration of Commonly Used Depression Scales Using Rasch Analysis. J Natl Compr Canc Netw. 2015;13:1203–1211. 26483060 10.6004/jnccn.2015.0149

[72] Olino TM, Lan Y, McMakin DL, Forbes EE, Seeley JR, Lewinsohn PM, et al Comparisons Across Depression Assessment Instruments in Adolescence and Young Adulthood: An Item Response Theory Study Using Two Linking Methods. J Abnorm Child Psychol. 2013;41:1267–1277. doi: 10.1007/s10802-013-9756-6 23686132 10.1007/s10802-013-9756-6 PMC3795839

[73] Alexandrowicz RW, Jahn R, Friedrich F, Unger A. The importance of statistical modelling in clinical research. Comparing multidimensional Rasch-, structural equation and linear regression models for analyzing the depression of relatives of psychiatric patients. Neuropsychiatr. 2016;30:92–102. doi: 10.1007/s40211-016-0180-3 27294269 10.1007/s40211-016-0180-3 PMC4917596

[74] Linacre JM. Sample Size and Item Calibration Stability. RMT. 1994;7:4 p.328.

[75] World Health Organization. Schedules for Clinical Assessment in Neuropsychiatry (SCAN). American Psychiatric Publishing, Incorporated; 1994.

[76] Wing JK, Babor T, Brugha T, Burke J, Cooper JE, Giel R. SCAN: Schedules for clinical assessment in neuropsychiatry. Arch Gen Psychiatry. 1990;47(6):589–593. 2190539 10.1001/archpsyc.1990.01810180089012

[77] World Health Organization. International classification of mental and behavioural disorders (ICD-10) Geneva: WHO; 1992.

[78] Masters GN. A Rasch model for partial credit scoring. Psychometrika. 1982;47(2): 149–174.

[79] Rijmen F, Briggs D. Multiple person dimensions and latent item predictors In: DeBock P, Wilson M, editors. Explanatory Item Response Models. New York: Springer; 2004 p. 247–265.

[80] Adams RJ, Wilson M, Wang WC. The multidimensional random coefficients multinomial logit model. Appl Psychol Meas. 1997;21(1):1–23.

[81] Holland PW, Wainer H, editors. Differential Item Functioning. Hillsdale: Lawrence Erlbaum; 1993.

[82] Wright BD, Masters GN. Rating Scale Analysis. Rasch Measurement. Chicago: MESA; 1982.

[83] Wright BD, Stone MH. Best Test Design. Rasch Measurement. Chicago: MESA; 1979.

[84] Smith AB, Rush R, Fallowfield LJ, Velikova G, Sharpe M. Rasch fit statistics and sample size considerations for polytomous data. BMC Med Res Methodol. 2008;8(1):33. 18510722 10.1186/1471-2288-8-33 PMC2440760

[85] Adams RJ. Reliability as a measurement design effect. Stud Educ Eval. 2005;31:162–172.

[86] Mislevy RJ, Beaton AE, Kaplan B, Sheehan KM. Estimating population characteristics from sparse matrix samples o f item responses. J Educ Meas. 1992;29:133–161.

[87] Akaike H. Information theory and an extension of the maximum likelihood principle In: Petrov BN, Csaki F, editors. Second International Symposion on Information Theory. Akadémiai Kiádó; 1973 p. 246–281.

[88] Sugiura N. Further analysts of the data by Akaike's information criterion and the finite corrections: Further analysts of the data by akaike's. Commun Stat Theory Methods. 1978;7(1):13–26.

[89] Hurvich CM, Tsai CL. Regression and time series model selection in small samples. Biometrika. 1989;76(2):297–307.

[90] Schwarz G. Estimating the dimension of a model. Ann Stat. 1978;6(2):461–464.

[91] Sclove SL. Application of model-selection criteria to some problems in multivariate analysis. Psychometrika. 1987;52:333–343.

[92] Bozdogan H. Model selection and Akaike's information criterion (AIC): The general theory and its analytical extensions. Psychometrika. 1987;52(3):345–370.

[93] Wald A. Tests of statistical hypotheses concerning several parameters when the number of observations is large. Trans Am Math Soc. 1943;54(3):426–482.

[94] R Core Team. R: A language and environment for statistical computing, Vienna: R Foundation for Statistical Computing; 2013 Available from: www.R-project.org.

[95] Robitzsch A, Kiefer T, Wu M. TAM: Test analysis modules. R package version 2.0–37 [software]. 2017. Available from: cran.R-project.org/package=TAM

[96] Boone WJ. Understanding person measures In: Boone WJ, Staver JR, Yale MS. Rasch analysis in the human sciences. Dordrecht: Springer; 2014 p. 69–91.

[97] Shafer AB. Meta-analysis of the Factor Structures of Four Depression Questionnaires: Beck, CES-D, Hamilton, and Zung. J Clin Psychol. 2006;62:123–146. doi: 10.1002/jclp.20213 16287149 10.1002/jclp.20213

[98] Alexandrowicz R, Weiss M, Marquart B, Wancata J. The validity of a two-step-screening procedure for depression. Psychiat Prax. 2008;35(6):294–301. 10.1055/s-2008-1067335 18504689

[99] Arroll B, Khin N, Kerse N. Screening for depression in primary care with two verbally asked questions: cross sectional study. BMJ. 2003;327(7424):1144–1146. doi: 10.1136/bmj.327.7424.1144 14615341 10.1136/bmj.327.7424.1144 PMC261815

[100] Spitzer RL, Williams JB, Kroenke K, Linzer M, Verloin deGruy F, Hahn SR, et al Utility of a new procedure for diagnosing mental disorders in primary care: the PRIME-MD 1000 study. JAMA. 1994;272(22):1749–1756. 7966923

[101] Bond TG, Fox CM. Applying the Rasch Model Fundamental Measurement in the Human Sciences. 3rd ed. New York: Routledge; 2015.

[102] Rasch G. An Individualistic Approach to Item Analysis In: Lazarsfeld PF, Henry NW, editors. Readings in Mathematical Social Science. Cambridge: The M.I.T. Press; 1966 p. 89–107.

[103] Rasch G. An informal report on the present state of a theory of objectivity in comparisons [Internet]. Universitetets Statistiske Institut; 1966 [cited 2017 Jun 17]. Available from: www.rasch.org/memo1966.pdf

