# Supplementary material for: Assessing the dimensionality of the CES-D using multi-dimensional multi-level Rasch models
Source: PLoS One. 2018 May 25;13(5):e0197908. doi: 10.1371/journal.pone.0197908 (PMC5969764; doi:10.1371/journal.pone.0197908)
Supplement: S1 File — (PDF) [file pone.0197908.s001.pdf]

The following remarks complement the discussion of the article

Assessing the dimensionality of the CES-D using multi-dimensional multi-level Rasch Models

Rainer W. Alexandrowicz, Rebecca Jahn, Johannes Wancata

Comparing Figs 1 and 3, we find the category thresholds of the four-dimensional model covering a broader range of values than in the one-dimensional approach. Hence, the subscale distinction allows for a better discrimination of the respondents. Correlations among the four subscales varied considerably ranging from 0.31 (I/IV) to 0.88 (II/III). Especially subscale IV, *Interpersonal Difficulties*, had weak correlations with the other subscales and correlations between Subscale I, *Positive Affect*, and the other subscales were just mediocre. Therefore, our recommendation is: Researchers applying the CES-D should prefer interpreting the subscale scores either separately or, if a total score is required, use only the subscales II, *Negative Affect* and III, *Somatic Symptoms* to compute the score. Contingently, subscale I could be taken as well, but the two items of subscale IV cannot be recommended due to the problems arising from its shortness. The present study is based on data from the general population, therefore no statements about the usefulness of subscale IV in specific populations (i.e. focussing on certain diagnoses) can be made.

In Fig 3, we found the person parameters of subscale II, *Negative Affect,* to cover a wider range on the latent depression scale compared to the other three subscales. A possible explanation could be the presence of related symptoms (to a certain degree) in the general population without meeting the diagnosis criteria for depression. For example, items like 14 ("I felt lonely"), 18 ("I felt sad"), or 10 ("I felt fearful") could very well apply to common events of everyday life and do not necessarily constitute an indication of depression in a clinical sense. These items could be considered not sufficiently specific to allow for differentiating clinically relevant depressive symptoms from common experiences. This could be regarded as a weakness of the CES-D.

Several studies reported different sum-scores for males and females or differing age groups [1,2]. Unfortunately, until now it is not yet clear if this is due to a differing prevalence or to a differing response behavior. Therefore, we performed a DIF analysis with respect to these two variables. This analysis revealed further interesting phenomena: Regarding gender, we find generally low effects, however, some of which were significant. Again because of the large sample size significance must be considered with caution not necessarily reflecting substantive differences. Therefore, we will only focus on the largest effects. These were most notable for items 9 (failure) and 17 (cry). The latter appears in line with existing

stereotypes, but such an interpretation would require further exploration, for example using a mixed-methodology study design. We also found several DIF effects for the depression split. First of all, the DIF analysis revealed an over-all effect, which is meaningful from a substantive point of view: Depressed respondents are generally more likely to endorse items expressing depressive symptoms or disagree to items expressing positive feelings. Regarding the results on item level, our findings for subscale I (Positive Affect) are: Item 4 (good) had a comparably large (and significant) DIF, item 12 (happy) had a weak (but significant) effect (item 8 could not undergo this analysis step). Considering that these are the reversed items, we probably face again a consequence of the aforementioned problem with reversed item wording, occurring even stronger for depressed respondents. These items were more difficult to (not) endorse for depressed respondents, even after taking a general tendency of depression into account. As has been worked out impressively in previous studies, it makes a difference to say “no” to “I feel good” rather than to say “yes” to “I feel bad” [3] or “I am not guilty” vs. “ I am innocent” [4]. Thus, the present study provides further evidence against reversed item wording, which has been revealed problematic from a psychometric point of view in several studies [5,6].

Subscale I, *Positive Affect*, deserves further consideration: The category thresholds were (in both the one- and the four-dimensional model) remarkably close to each other in items 4 (good) and 8 (hopeful). Hence, these two items did not discriminate very well in either approach. They also show significantly deviating infit indices again indicating poor item functioning. At first sight, one might conclude that the respondents perceived them in a more dichotomous fashion, i.e., only the lowest and the highest categories appeared relevant to them, while regarding the two middle categories as not meaningful. However, looking at the response frequencies in detail this was not the case. While the two remaining items of this subscale (12 and 16) show increasing frequencies from response category 0 to 3 (item 12: 8%–12%–36%–43%; item 16: 7%–15%–27%–50%), the respective percentages of the suspicious items were much more balanced (item 4: 22%–8%–16%–54%; item 8: 21%–19%–24%–35%). Considering that the four items of this subscale are all reversed (i.e., category 3 indicating well-being), we might face the problem that some respondents might have overlooked the reversion thus erroneously choosing category 0 for well-being like in the previous items. Corroborating this interpretation, the affected items (4 and 8) are the first two reversed items in the questionnaire. This issue has to be addressed in future analyses.

But aside of the rather technical reversed wording effect, we also have to take substantive aspects associated to depression into account. Items 4 (“I felt that I was just as good as other people”), 8 (“I felt hopeful about the future”), 13 (“I talked less than usual”), and 15 (“People were unfriendly”) were more difficult to endorse for respondents fulfilling the depression criteria. These effects could be related to increased occupation with oneself, when in a depressed mood, and subsequent tendencies of social withdrawal. Hence, such items might

not appear to be relevant to them. In contrast, more easily endorsed were items 6 (“I felt depressed”), 9 (“I thought my life had been a failure”), 10 (“I felt fearful”), 14 (“I felt lonely”), and 7 (“I felt that everything I did was an effort”). These items relate to core symptoms of depression and might therefore attract depressed respondents unevenly more than those without depression. Nevertheless, most of the DIF effects were comparably small, thus posing no severe obstacle for using the CES-D.

References

1. Riediger M, Linden M, Wilms HU. (1998). Die deutsche Version der CES-D als Instrument der gerontologischen Forschung. Z Klin Psychol Psychiatr Psychother; 46(4): 344–364
2. Stein J, Luppa M, Mahnke J et al. (2014). Depressionsscreening am Telefon mittels der Allgemeinen Depressionsskala (ADS). Psychiatr Prax; 41(03): 135-141
3. Enos MM. (2000). Just Say “No!”. The Impact of Negation in Survey Research. PM. 2000;3(1):34-39.
4. Mayo R, Schul Y, Burnstein E. “I am not guilty” vs. “I am innocent”: Successful negation may depend on the schema used for its encoding. J Exp Soc Psychol. 2003;40:433-449.
5. Weijters B, Baumgarnter H. Misresponse to Reversed and Negated Items in Surveys: A Review. J Mark Res 2012; XLIX:737–747.
6. Cabooter E, Weijters B, Geuens M, Vermeir I. Scale format effects on response option interpretation and use. J Bus Res. 2016; 69(7): 2574–2584.
